# Prediction of alternative pre-mRNA splicing outcomes

**DOI:** 10.1038/s41598-023-47348-6

**Published:** 2023-11-15

**Authors:** Rayan Najjar, Tomas Mustelin

**Affiliations:** Division of Rheumatology, Department of Medicine, University of Washington, 750 Republican Street, Seattle, WA 98109 USA

**Keywords:** Transcriptomics, RNA splicing, Genome informatics, Software

## Abstract

To understand the biological impact of alternative pre-mRNA splicing, it is vital to know which exons are involved, what protein domains they encode, and how the translated isoforms differ. Therefore, we developed a computational pipeline (RiboSplitter) focused on functional effect prediction. It builds on event-based alternative splicing detection with additional filtering steps leading to more efficient statistical testing, and with detection of isoform-specific protein changes. A key methodological advance is reading frame prediction by translating exonic DNA in all possible frames, then finding a single open reading frame, or a single frame with matches to known proteins of the gene. This allowed unambiguous translation in 93.9% of alternative splicing events when tested on RNA-sequencing data of B cells from Sjögren’s syndrome patients. RiboSplitter does not depend on reference annotations and translates events even when one or both isoform(s) are novel (unannotated). RiboSplitter’s visualizations illustrate each event with translation outcomes, show event location within the gene, and align exons to protein domains.

## Introduction

Alternative splicing of primary transcripts contributes greatly to proteome diversity and has broad biological implications in health^1^ and disease^2–4^. RNA sequencing technology has enabled quantifying alternative splicing events (will be referred to as “events”) and multiple in silico tools have been developed to analyze alternative splicing^5^. However, most existing tools focus on detection, quantification, and differential statistical testing of splicing events^6^, leaving the prediction of structural and functional protein changes underdeveloped. To fill this gap, we developed RiboSplitter, which focuses on the biological consequences of these splicing events at the protein level, revealing the location of the involved exons in relation to other exons of the gene and predicting the resulting protein changes by overlaying the translation product with domain annotations.

Many alternative splicing detection tools only identify events with known junction annotations^5^. Because the exon–intron annotation of the reference genome likely remains incomplete, and because disease states can lead to varying levels of unannotated (novel) and perhaps aberrant splicing events, it is essential to have tools that detect both annotated and unannotated splicing events, not to miss potentially important biology. Among the tools that can detect unannotated events, additional methods to separate noise from signal are needed to add confidence in the detected events. For example, a single read spanning a splicing junction should not yet be considered enough evidence to call a splicing junction, because such a read may represent sequencing and/or alignment error. For this reason, we used SplAdder^7^ to develop our analysis pipeline. SplAdder is an event-based tool that detects both annotated and unannotated splicing events, and applies filtering of alignment reads with 4 levels of confidence based on alignment quality, number and overlap of reads spanning a splicing junction, and intron size. These criteria allow identifying unannotated events with high confidence.

RiboSplitter’s analytical pipeline performs event filtering, statistical testing, multiple comparison adjustment, reading frame prediction, protein translation, detection of amino acid differences between isoforms, and creation of event genomic names. All of this is performed with one function call, to improve usability. Next, RiboSplitter generates figures that show event details and alignment to protein domains, e.g. this allows prediction of which protein domain would be missing if an exon is skipped.

RiboSplitter introduces multiple advances in alternative splicing research. The filtering steps we employ improve statistical efficiency and avoid performing hypothesis testing on thousands of events that are in non-coding RNA or are unlikely to show differential expression (too many missing values and/or very low variability). We used a beta binomial model with two distributions for disease and control that better approximate biological differences. We show that our filtering steps and model result in improvement in differential event detection, compared to SplAdder’s methodology. Additionally, RiboSplitter predicts reading frames for the majority of events, allowing protein translation to compare differences between isoforms at the amino acid level. Bisbee^8^ predicts protein translation of alternative splicing events only when at least one isoform is annotated. RiboSplitter overcomes this limitation and translates all types of events even when they are completely novel (unannotated). Finally, RiboSplitter aligns event exons to protein domains, unlike Bisbee, allowing prediction of functional impact of alternative splicing on protein domains.

RiboSplitter is flexible. We used SplAdder’s output as a starting point for our analytical pipeline. However, other event-based alternative splicing detection software can be used instead. RiboSplitter takes sample level percent-spliced in (PSI) and reads supporting isoforms data, plus event genomic positions. Therefore, if these can be generated by an alternative splicing detection tool, including from long read and single cell tools, then RiboSplitter can be used for downstream analysis.

## Results

### RiboSplitter pipeline

**Figure 1:**
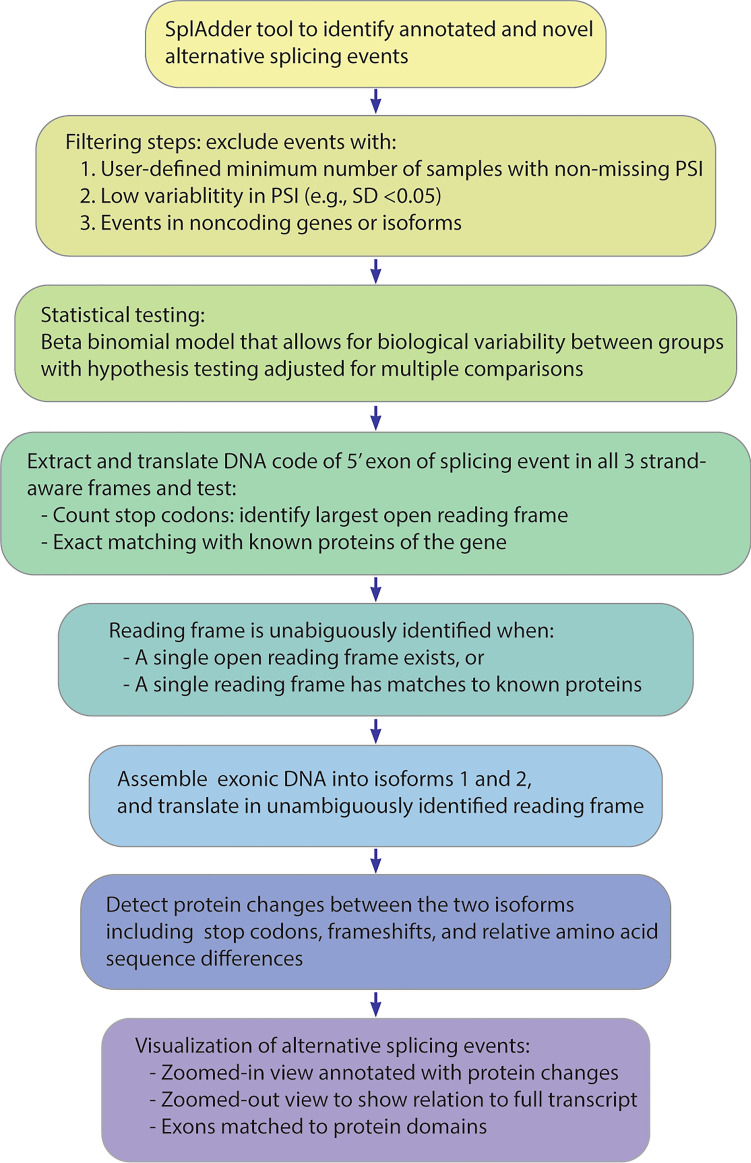
Flowchart of RiboSplitter analysis pipeline. PSI = Percent-spliced in.

Figure 1 shows a flowchart of the pipeline (see “Methods” for details). The key insight in RiboSplitter is to initially go small and predict the reading frame of the first 5´ exon of the splicing event, instead of trying to translate the full isoform. This is because the full isoform contains more exons with higher chances of encountering multiple stop codons. Predicting the reading frame is the main challenge in translating alternative splicing events. This is because of multiple reasons: event-based splicing tools focus on 3–4 exons, the presence of multiple transcripts per gene with potentially different starting exons, the incompleteness of our transcript and exon annotations, and the fact that disease states can lead to novel splicing events with novel sequences. We overcome these challenges by focusing on the first 5´ exon of an alternative splicing event which is shared among the two isoforms of the event. Then, we use two main methodologies to identify the reading frame. We translate the exonic DNA code to protein in all 3 possible frames (strand-aware) to find open reading frames, and we use exact matching of the translated peptides to known protein products of the gene. We consider the reading frame to be unambiguously identified if a single reading frame results in an open reading frame or matches to a known protein. Once the reading frame is identified, we assemble the exonic DNA sequences and translate the two isoforms, allowing identification of stop codons, frameshifts, and altered amino acid sequences.

### Visualizations

RiboSplitter produces 3 types of visualizations in order to help understand the effects of alternative splicing. One zoomed-in view of the event shows the two isoforms with genomic coordinates, and is annotated with stop codons and frameshifts. In order to show altered amino acid sequences, we color exons light blue if the expected translated peptide is the same in the two isoforms, and light green if the expected translated peptide is different. Next to the zoomed-in figure, a jitter plot shows PSI values for all samples grouped as cases and controls. In order to interpret the consequences of an alternative splicing event, we need to see which exons are involved within the transcript. Therefore, we visualize a zoomed-out view of the event with the closest matching transcript based on the first and last exons of an alternative splicing event (the two exons shared between the two isoforms). Finally, RiboSplitter produces figures of exons aligned to protein domains from the InterPro database^9^. InterPro entries include protein domains, families, repeats, sites (active, binding, conserved), and other unintegrated entries.

### RiboSplitter’s output

Besides the illustrative figures, RiboSplitter outputs an event-level dataset with gene and event IDs, event IDs based on genomic coordinates, average PSI per group, *p* values, and other descriptive variables. Protein prediction variables include the amino acid sequences of both isoforms, and which parts of the sequences are the same versus different between the two isoforms, as well as information about stop codons and frameshifts.

### Application to circulating B cells from Sjögren’s syndrome patients

We applied RiboSplitter to RNA sequencing data of peripheral blood CD19^+^ B cells from patients with primary Sjögren disease (publicly available, GEO GSE199868). After alignment to the reference genome (GRCh38.p13), we used SplAdder to identify annotated and novel alternative splicing events. There were 91,310 high confidence (based on SplAdder’s criteria) alternative splicing events in 6383 genes.

### Statistical testing

**Figure 2:**
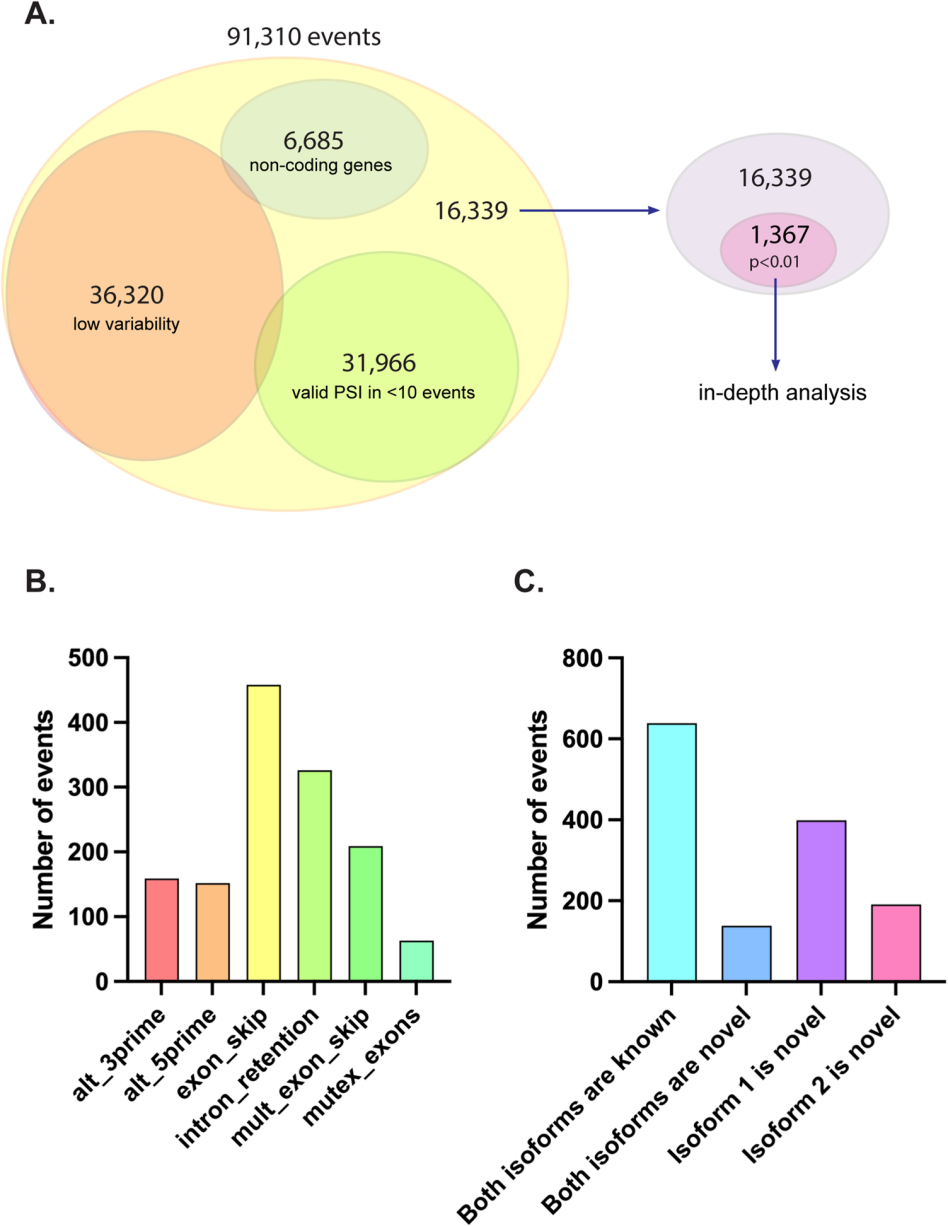
Frequencies of filtering steps and types of events and isoforms in alternative splicing of Sjögren B cells. (**A**) Application of RiboSplitter’s filtering steps on 91,310 high confidence alternative splicing events. (**B**) Frequencies of event types. (**C**) Frequencies of events by number of novel (unannotated) isoforms. PSI = percent-spliced in. *p* values were computed using beta binomial models.

After applying RiboSplitter’s filters (Fig. 2A), statistical testing yielded 1367 alternative splicing events that were statistically different in Sjögren B cells compared to controls (alpha = 0.01). We compared our differential testing to that of SplAdder’s using default settings. SplAdder uses a generalized linear model with negative binomial distribution to model junction read counts, and controls for gene expression. In contrast, our approach was to model PSI, which is unaffected by differential gene expression. For example, if a gene is overexpressed in Sjögren over control without a change in splicing, then we would observe higher counts of both isoforms without a change in PSI.

SplAdder identified 824 statistically significant events but 244 of them had few samples (≤ 4 per group) including multiple events with a single sample in the Sjögren or control groups. An additional 108 events had < 10 samples per group, and 202 were from noncoding genes and transcripts.

Out of 202 events identified by RiboSplitter as statistically significant with delta average PSI of 0.2 or higher, SplAdder only flagged 38 as statistically significant. In addition to having high delta average PSI, the 164 events missed by SplAdder had high support per event (median 1279.5 reads, range, 341–26,872).

SplAdder ran hypothesis testing for 72,448 events, while RiboSplitter ran hypothesis testing on 16,339 events because we excluded the thousands of events that were in noncoding genes, had a lot of missing values, or had very low overall variability in PSI. A more aggressive and statistically sound filtering is needed to avoid the ‘cost’ of excessive multiple testing.

### Reading frame prediction

RiboSplitter unambiguously identified the reading frame in 1283 out of the 1367 statistically significant alternative splicing events (93.9%). The two methodologies of identifying a single open reading frame and peptide matching to known proteins of the genes predicted the same reading frame in 674 events, and another 532 events by either method (Table 1). The two methods never predicted conflicting reading frames. In a subset of events, the two methods provided ambiguous results, meaning two or three frames resulted in open reading frames or matched to existing proteins. To help resolve this, we checked if testing the full isoform for a single open reading frame would help, which clarified 77 additional frames. The percentages of unambiguously identified reading frames were similar across events with 0, 1, or 2 novel (unannotated) isoforms (range 93.4–94.4%).

**Table 1:** Reading frame predictions based on two methodologies.

Open reading frame method	Peptide matching method				
	Frame 1	Frame 2	Frame 3	X	Total
Frame 1	330	0	0	50	380
Frame 2	0	140	0	42	182
Frame 3	0	0	204	78	282
X	163	74	125	161	523
Total	493	214	329	331	1367

*X* unknown frame.

### Sjögren B cells alternative splicing

The 1367 differential events detected by RiboSplitter in Sjögren B cells versus control occurred in 904 protein-coding genes (Supplementary Table 1), with a range of 1–12 events per gene. The most common types were exon skips (458, 33.5%) and intron retentions (326, 23.9%) (Fig. 2B). Both isoforms were novel in 138 events (10.1%), and 590 events had one novel isoform (43.2%) (Fig. 2C). Events with bigger delta average PSI (larger effect size) between Sjögren and control are more likely to be biologically meaningful. There were 746 events (54.6%) with delta average PSI of ≥ 0.1, and 206 (15.1%) events with delta average PSI of ≥ 0.2. The top 90 events by delta average PSI are illustrated in Supplementary Figs. 1–9.

**Figure 3:**
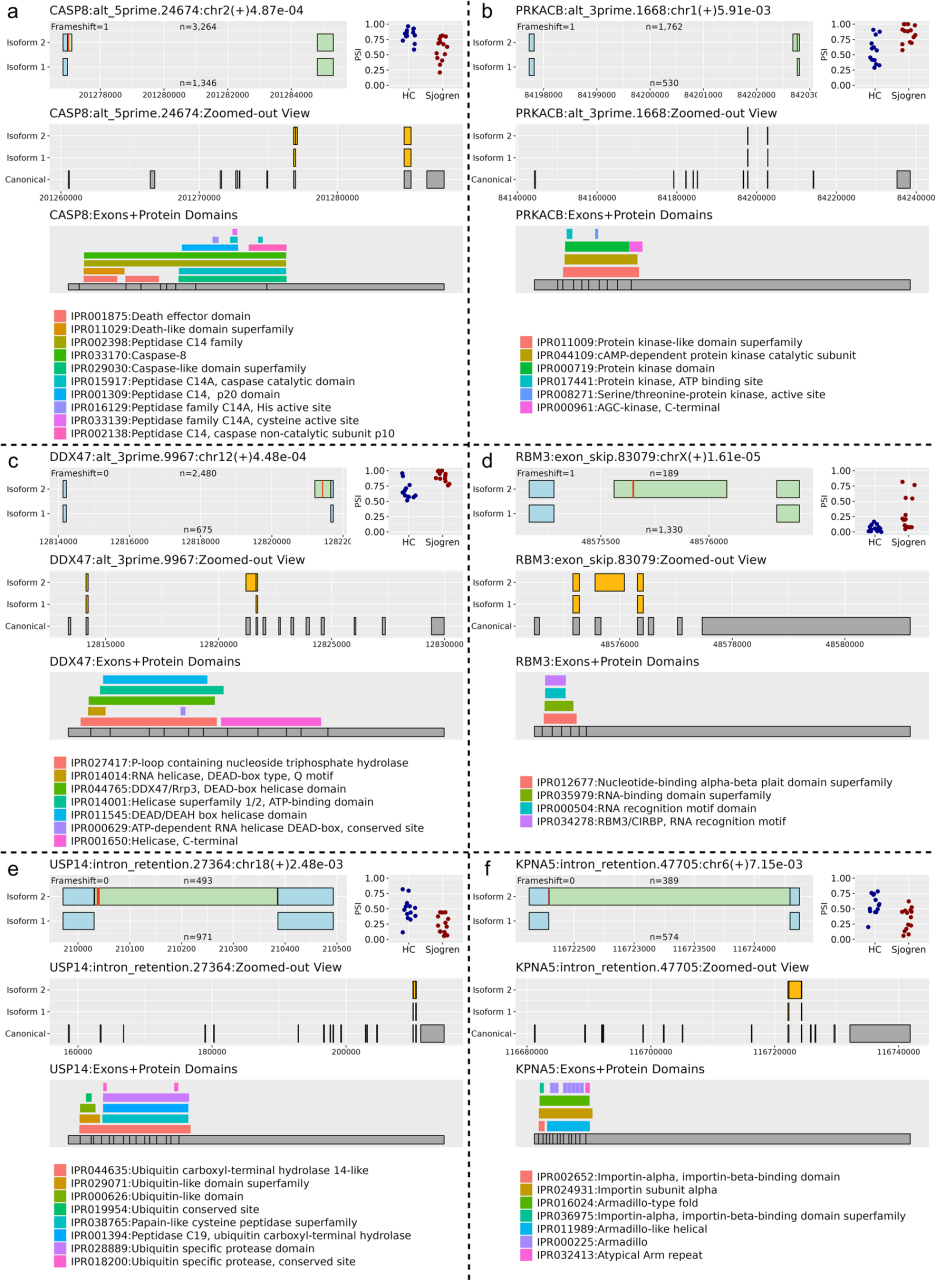
Selected alternative splicing events in Sjögren B cells versus control. Top left figure shows a zoomed-in view of the alternative splicing event. The title includes gene name, event ID, chromosome, strand, and adjusted *p* value. n is the number of reads supporting isoform 1 or 2. Red lines represent the first stop codon per isoform. Light blue exons indicate same protein product, while light green indicate altered peptides when translated. Top right figure shows percent-spliced in (PSI) of isoform 2 in Sjögren and healthy control (HC). Middle figure shows a zoomed-out view of the alternative splicing event with parallel representation of exon–intron structure of the canonical transcript. Bottom figure shows collapsed exons of the transcript aligned with protein domains of the gene. Panels (**a**), (**b**), (**c**), (**d**), (**e**), and (**f**) illustrate six alternative splicing events in different genes.

We discovered an event in the caspase 8 gene that resulted in a longer exon 7, introducing a stop codon and a frameshift, and leading to a truncated protein lacking the catalytic domain (Fig. 3a). The truncated protein was less prevalent in Sjögren B cells, favoring increased apoptosis. A very similar event was reported in 2001^10^ affecting exon 8 of the same gene. We investigated if the two events were, in fact, the same event by studying the primer sequence of fragment C (GCATTAGGGACAGGAATGGA) from the original paper, where a 2nd larger band was seen by RT-PCR. We performed exact matching of the fragment C sequence and found that it is part of exon 7, not exon 8 as previously described. Additionally, the originally sequenced intronic insert matches to the intron between exons 7–8, not 8–9. Finally, our translated truncated peptide matches exactly what was previously reported (TVEPKREK*, Table 2) that ends with a stop codon. Considering which exons encode for which protein domains (Fig. 3a), one can see that a stop codon right after exon 7 would truncate the protein shortly after the death-effector domains, while a stop codon right after exon 8 would result in a protein that still contained the catalytic sites.

**Table 2:** Output from RiboSplitter showing protein changes for 6 selected alternative splicing events.

Gene	Event ID	Same start AA	AA different in isoform 1	AA different in isoform 2	Same end AA
*CASP8*	alt_5prime.24674	TLDKVYQMKSKPRGYCLIINNHNFAKAREKVPKLHSIRDRNGTHLDAG	ALTTTFEELHFEIKPHDDCTVEQIYEILKIYQLMDHSNMDCFICCILSHGDKGIIYGTDGQEAPIYELTSQFTGLKCPSLAGKPKVFFIQACQGDNYQKGIPVETDSEEQPYLEMDLSSPQTRYIPDEADFLLGMATVNNCVSYRNPAEGTWYIQSLCQSLRERCP	TVEPKREK*	
*PRKACB*	alt_3prime.1668	VTDFGFAKRVKGRTWTLCGTPEYLAPEIILSK	TNQFRFMKRLFLE	GYNKAVDWWALGVLIYEMAAGYPPFFADQPIQIYEKIVSGK	
*DDX47*	alt_3prime.9967	GVTDVLCEACDQLGWTKPTKIQIEAIPLALQ		GRDIIGLAETGSGKTGAFALPILNALLETPQRLFALVLTPTRELAFQISEQFEALGSSIGVQSGKCLRGKGS*	AVIVGGIDSMSQSLALAKKPHIII
*RBM3*	exon_skip.83079	LELPCPLKKESSSWEGSTLTPTSRHWKTTSAVSDLSLR	SLDGRQIRVDHAGKSARGTRGGGFGAHGRGRSYSR	WSLSRTGRLSGPGVLVSSPSPTQSMLQLP*	
*USP14*	intron_retention.27364	SDKKSSPQKEVKYEPFSFAD		GK*	DIGSNNCGYYDLQAVLTHQGRSSSSGHYVSWVKRKQ
*KPNA5*	intron_retention.47705	VSPCLNVLSRLLFSSDPDVLADVCWALSYLSDGPNDKIQAVIDSGVCRRLVELLM		*	HNDYKVVSPALRAVGNIVTGDDIQTQ

Amino acid sequences (AA) that are the same at the start and end of isoforms 1 and 2, and sequences that are different in one isoform relative to the other. *Stop codon.

Another example of alternative splicing occurred in a gene encoding a protein kinase where an alternative 3´ site in the 8th exon of the protein kinase *PRKACB* resulted in a frameshift in the last 3 exons. This isoform was more prevalent in Sjögren B cells (Fig. 3b). The frameshift would alter the amino acid sequence of the cAMP-dependent protein kinase catalytic subunit and the AGC-kinase at the C-terminal. This gene is involved in cAMP signaling.

In another illustration of what RiboSplitter can detect, we observed that alternative splicing affected several genes encoding proteins acting on RNA. For example, *DDX47*, an RNA helicase, had multiple alternative splicing events including one that was more common in Sjögren B cells where an intronic region after exon 3 is spliced in resulting in a stop codon that would lead to a truncated protein lacking the ATP-dependent RNA helicase DEAD-box and C-terminal domain (Fig. 3c). Another example is an isoform more prevalent in Sjögren B cells with an intronic region spliced in after exon 3 of *RBM3*. This resulted in a stop codon that would disrupt the RNA binding domain of the protein (Fig. 3d).

An example of an intron retention event that was detected by our pipeline was an event in *USP14* (ubiquitin specific peptidase 14) which is a deubiquitinating enzyme. The retained intron was more common in healthy B cells, and introduced a stop codon in the C-terminus disrupting the last 2 exons that encode for a ubiquitin specific protease conserved site (Fig. 3e). Another example of intron retention was observed in *KPNA5*, which is involved in protein transport into the nucleus, where a retained intron introduced a stop codon disrupting the armadillo repeat domains and the atypical arm repeat at the C terminus (Fig. 3f). These examples illustrate the utility of RiboSplitter in analyzing the outcomes of altered mRNA splicing.

### Software and usability

RiboSplitter was written in R language^11^ (v. 4.2.1) and uses the following R packages: tidyverse^12^ (v. 2.0.0), Biostrings^13^ (v. 2.66.0), biomaRt^14^ (v. 2.54.1), patchwork^15^ (v. 1.1.3), rhdf5^16^ (v. 2.42.1), and aod^17^ (v. 1.3.2). In addition, the pipeline uses BEDTools^18^ (v. 2.30.0). We used SplAdder to detect event-based alternative splicing events, but other tools can be used as well.

We have created a streamlined pipeline where the “ribosplitter” function runs the full pipeline including filtering, statistical testing, multiple comparison adjustment, reading frame prediction, protein translation, detection of amino acid differences between isoforms, and creation of genomic names for alternative splicing events.

RiboSplitter is fast. Using a server with CPU of 3.00 GHz and 186 GB RAM, it took 24 min to run RiboSplitter on the Sjögren B cells data.

## Discussion

We present RiboSplitter, an analysis pipeline that focuses on predicting the effects of alternative splicing events, an area that is underdeveloped in genomics. First, RiboSplitter runs filtering steps to exclude events that are noncoding, low-variability, or have excessive missing values, thereby allowing more efficient statistical testing. Second, RiboSplitter establishes methodologies to unambiguously detect the reading frames in alternative splicing events, even when one or both isoforms are unannotated. This allows translation and identification of protein changes for the majority of events. Third, RiboSplitter creates 3 types of visualizations to show details of the splicing events with annotations of protein changes, to locate the event within the larger transcript, and to align exons with their encoded protein domains.

We are aware of one other tool that performs predictions of protein changes in alternative splicing events. Bisbee^8^ predicts protein sequences from novel splicing events only if one isoform is in the reference annotation, while RiboSplitter does not rely on the reference annotation and therefore, can translate completely unannotated (novel) alternative splicing events. In the Sjögren B cell data, 10.1% of the statistically significant events were completely novel (both isoforms were unannotated). The percentage of completely novel events varies by disease, and was 23.4% in lupus RNA-sequencing data (unpublished). Additionally, Bisbee does not map protein domains to exons.

We applied our analysis pipeline to B cells from Sjögren syndrome and found numerous alternative splicing events with high delta average PSI. We were able to detect protein changes for the majority of these events. We showed that a previously described event in caspase 8 resulting in a truncated protein is a result of an alternative 5´ splice site causing a longer exon 7, not exon 8 as previously thought^10,19^. The isoform encoding the truncated protein was shown to be more prevalent in resting lymphocytes, which shifts to more expression of the intact isoform on activation^20^. This is similar to our finding of less expression of isoform 2 in B cells from Sjögren patients. Other studies focused on characterizing the presence and function of the isoform in undifferentiated neuroblastoma cancer^21^ and HTLV-infected adult T-cell leukemia^22^.

RiboSplitter is flexible. We used SplAdder to develop this methodology. However, any event-based alternative splicing detection tool can be used. Two datasets are needed as input: one event-level dataset with genomic coordinates of exons, unique event IDs, and gene names; and a second sample-level dataset with the number of reads supporting isoforms 1 and isoform 2 for each event plus the PSI. RiboSplitter is also flexible in that one can choose to use the differential statistical testing in RiboSplitter or use the native method of the splicing detection tool. Additionally, users can control the initial filtering steps.

In summary, RiboSplitter introduces methodological advances in the study of alternative splicing consequences at the protein level. It builds on event detection tools to perform downstream analysis predicting translation products for the majority of events, even if completely novel. Finally, the pipeline is flexible, streamlined, and fast.

## Methods

### Data

Publicly available Paired-end RNA sequencing data of circulating CD19^+^ B cells from primary Sjögren syndrome (n = 14) and healthy controls (n = 13) were downloaded from NCBI (GEO accession GSE199868).

### Alignment to reference genome and alternative splicing detection

After QC of sequencing quality, FASTQ files were aligned to the reference genome (GRCh38.p13) using STAR^23^ (v. 2.7.10b) with two-pass mode which improves detection of reads mapping to novel splicing junctions. After post-alignment QC, sorted and indexed BAM files were used to identify alternative splicing events by SplAdder^7^ (v. 3.0.3) which uses alignment and annotation data to create splicing graphs that are enriched with splicing events based on the provided RNA-seq data. SplAdder detects both annotated and novel splicing events and is capable of detecting 6 types of events: exon skip, alternative 3´/5´ splice site, intron retention, mutually exclusive exons, and multiple exon skip. SplAdder filters input alignment reads based on their quality and allows 4 levels of confidence levels, we ran the software with the highest confidence setting. Additionally, SplAdder uses event-specific criteria to confirm splicing events based on genomic coordinates and coverage, we only included confirmed events in our analysis.

### Types of events and nomenclature in SplAdder and RiboSplitter

A consistent nomenclature is essential to understand which isoform is more expressed in which group. SplAdder uses a naming convention where the longer isoform (the one with more exons) is isoform 2 and the shorter one is isoform 1. This works well for 5 of the 6 event types. However, either of the mutually exclusive exons can be the longer one, or they can have the same length. Therefore, for mutually exclusive events, we consistently considered isoform 1 to be the isoform with the first mutually exclusive exon based on genomic coordinates, to avoid confusion.

The percent spliced-in (PSI) is the number of reads supporting one isoform divided by the sum of reads supporting isoform 1 + isoform 2. We consistently calculate PSI for isoform 2, so that PSI values approaching 1 indicate higher expression of isoform 2 and values approaching 0 indicate higher expression of isoform 1. Consistent with SplAdder’s methodology, PSI was set to missing when the sum of reads supporting isoform 1 plus reads supporting isoform 2 per event per sample was < 10 to avoid unstable PSI estimates with low read counts. This cutoff can be easily changed in our pipeline depending on research goals (casting a wide net versus focusing on events with higher support).

### Filtering steps

We apply filtering steps before we run differential testing in order to improve statistical and computational efficiency. We exclude events with less than a minimum number of samples with valid PSI. We recommend allowing events to have few samples with missing PSI since this can happen for technical sequencing reasons (e.g. low coverage), or due to disease heterogeneity. We included events with ≥ 10 samples with valid PSI per group in the Sjögren B cells analysis. SplAdder’s differential testing methodology excludes events based on delta average PSI of cases and controls. In contrast, we filter events based on the standard deviation (SD) of PSI for the overall samples per event, which is a more agnostic way of filtering allowing variability to arise from within or from across groups. We used A cutoff of 0.05 in PSI SD in the Sjögren B cells analysis. Finally, RNA-sequencing data contain noncoding RNA, which undergoes even more extensive alternative splicing compared to coding RNA^4^, as it lacks selective pressure to maintain an open reading frame. Since we are interested in protein coding genes, we excluded splicing events from noncoding genes.

### Hypothesis testing

Alternative splicing events are non-negative counts data and can be thought of in terms of a binomial distribution, where there is a binary outcome of either isoform 1 or 2, with the total read count of isoforms as the coverage (number of trials), the Sjögren and control samples as different draws from the distribution, and PSI as the probability of success. Additionally, we need to allow probabilities in Sjögren and control groups to be different. Therefore, we implemented a beta binomial model with an overdispersion parameter which allows for biological variability between the groups. The model was fit using the “betabin” function from the R package aod (v. 1.3.2), as follows: betabin (cbind(y, n-y) ~ group, random =  ~ group, data = df, link = 'logit'), where the left-hand side represents the fixed effects, with y being the number of reads supporting isoform 2, and n being the sum of reads supporting isoform 1 and isoform 2. The modelled probability is y/n which is the PSI. The right-hand formula represents the overdispersion parameters (phi), with phi being different for levels of “group” (i.e. two beta distributions, one for the disease samples and one for the control samples). Finally, “df” is the data frame with these input parameters. We adjusted the two-sided *p* values for multiple comparisons using the Benjamini–Hochberg method^24^, and used an alpha = 0.01 as cutoff for statistical significance. We calculated delta average PSI for each event, which is the difference in average PSI between disease and control samples.

### Protein change prediction

We extracted DNA sequences of all exons in the identified splicing events using strand-specific genomic positions (chromosome number, start and end positions) using BEDTools^25^ (v. 2.30.0). To identify the correct reading frame, we focused on the first 5´ exon of each event, which is shared in both isoforms. We used two methodologies to detect the correct reading frame of this exon. First, exonic DNA code was translated in the 3 possible frames (DNA code was strand-aware), to look for a single open reading frame. Second, the translated code of the first 5´ exon in different reading frames was matched to known protein peptides, and matches were counted by reading frame. We considered a reading frame to be unambiguously identified either when there was a single open reading frame, or when peptides of one reading frame matched to known proteins, and the other 2 reading frames had zero matches. For events with still ambiguous reading frames, we counted stop codon of the full assembled exonic DNA in all reading frames. After a single reading frame is unambiguously identified, we assembled exons of each isoform of the alternative splicing event and translated their DNA sequence into amino acids. Protein changes were identified by comparing the two amino acid sequences including detection of stop codons, frameshifts introduced by middle exons, and other changes.

### Visualizations

The zoomed-in view of alternative splicing event was annotated with protein changes. The distance from the beginning of amino acid sequence to the first stop codon was converted to a genomic DNA coordinate to show their location on the exons. The same process was repeated to identify the locations of differences in amino acid sequences on the isoforms.

The zoomed-out view of alternative splicing events was created by matching the first and last exons of the event to the best fitting transcript of the gene. Transcript and exon information is acquired from Ensembl^26^ using the biomaRt (v. 2.52.0) package.

The protein domains figures were created by acquiring protein domains information from InterPro^7^. Since InterPro collates protein domain predictions from different sources, domains have slightly different start and stop predictions. Therefore, we collapsed same domain predictions into one, to avoid cluttered figures with too many predictions of the same domain. Protein domains distances were offset by the distance from transcription start site to translation start site based on information from Ensembl. Exons were collapsed removing introns from figure. Finally exons and domains were aligned and depicted in figures.

### Supplementary Information

Supplementary Figures.

Supplementary Tables.

## Data Availability

We used publicly available RNA sequencing data of circulating CD19^+^ B cells from patients with primary Sjögren’s syndrome and healthy controls (GEO accession GSE199868).

## Code availability

RiboSplitter’s code is publicly available at https://github.com/R-Najjar/RiboSplitter.
