# Supplementary material for: Prediction of alternative pre-mRNA splicing outcomes
Source: Sci Rep. 2023 Nov 15;13:20000. doi: 10.1038/s41598-023-47348-6 (PMC10651857; doi:10.1038/s41598-023-47348-6)

RiboSplitter: a pipeline for predicting the outcomes of alternative pre-mRNA splicing

By Rayan Najjar* and Tomas Mustelin

Division of Rheumatology, Department of Medicine, University of Washington, 750 Republican Street, Seattle, WA 99108
*Correspondence to Dr. Rayan Najjar MD, MPH, phone (206) 685-9557, e-mail: najjar@uw.edu

Extended Data Legends

Extended Data Fig. 1-3. Zoomed-in view of top 90 alternative splicing events by delta average percent spliced-in (PSI) of Sjögren vs control B cells. Figure titles include gene name, event type and ID, chromosome number, strand, and adjusted p value. In upper left corner, frameshift indicates a relative reading frame frameshift between the two isoforms. n is the number of reads supporting the isoform. Red lines represent the first stop codon in the isoform. Blue exons have the same amino acid codons when translated, while green exons have altered peptide sequences. Note that these color representations are approximate since codons can span splice junctions. Next to each splicing event representation is a jitter plot of PSI of isoform 2 in Sjögren and healthy controls (HC)

Extended Data Fig. 4-6. Zoomed-out view of top 90 alternative splicing events by delta average percent spliced-in (PSI) of Sjögren vs control B cells. Grey exons are the full exons of the transcript that best fits the alternative splicing event

Extended Data Fig 7-9. Protein domains aligned to exons of the top 90 alternative splicing events by delta average percent spliced-in (PSI) of Sjögren vs control B cells (68 unique genes).

Extended Data Figure 1

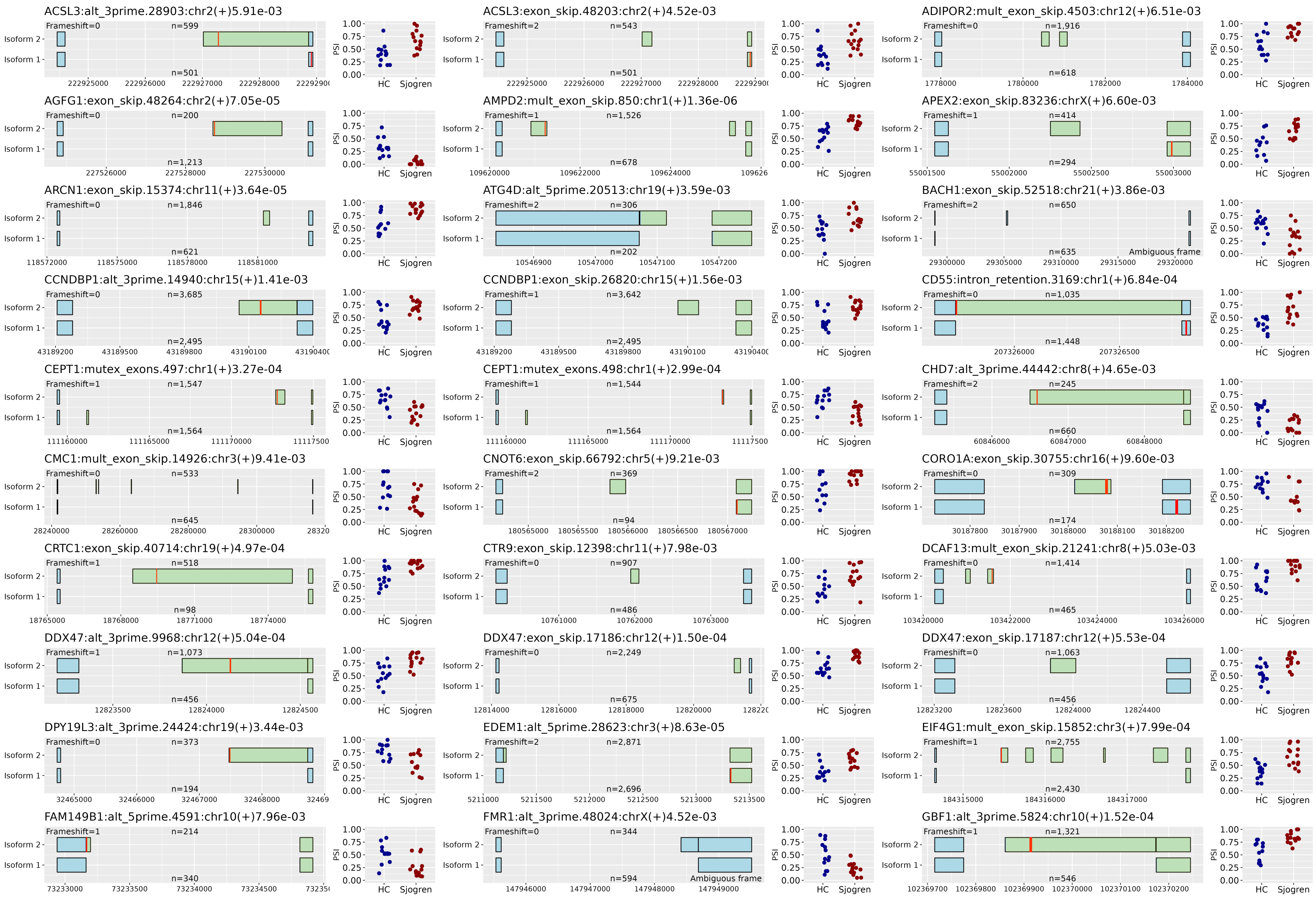

Extended Data Figure 2

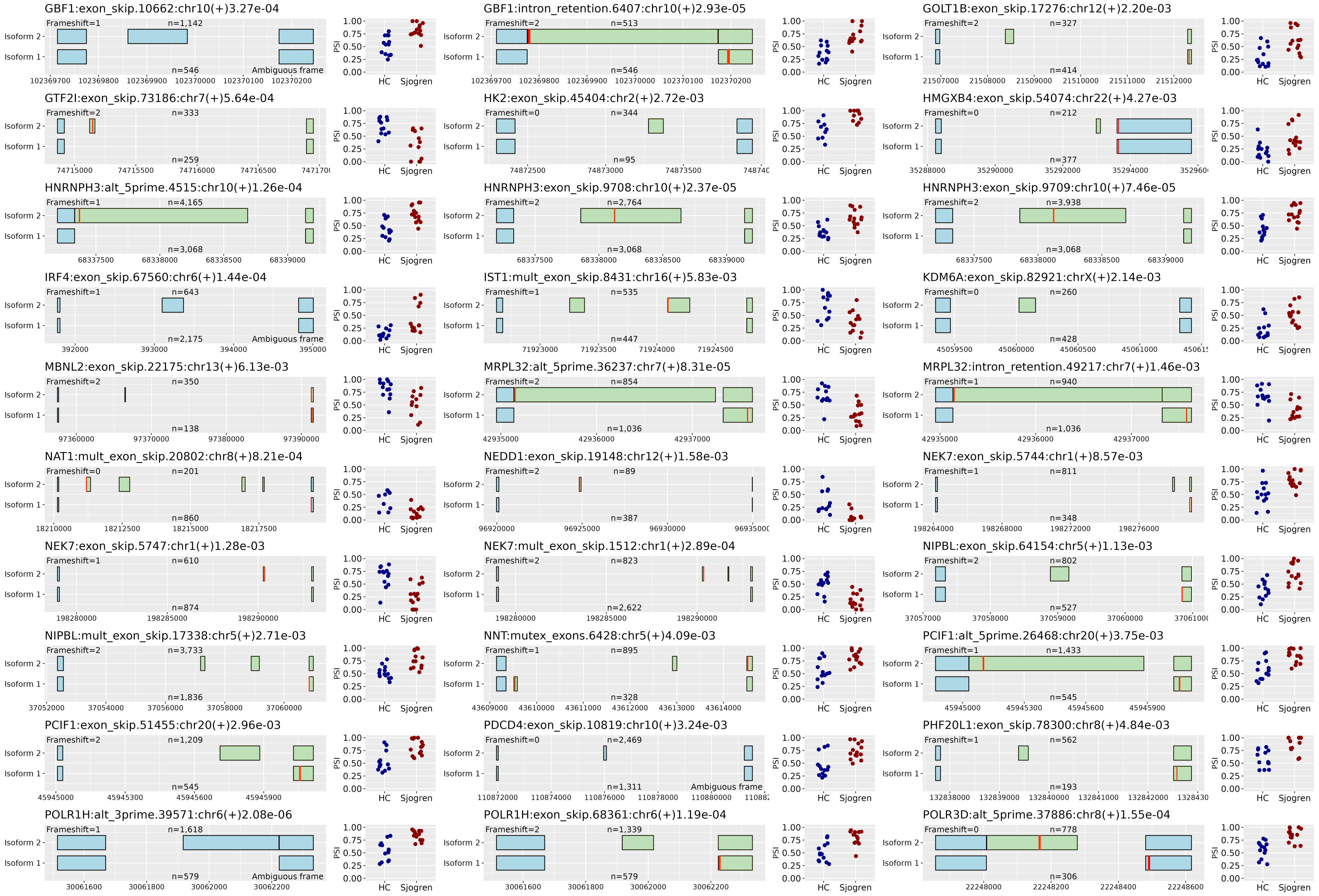

Extended Data Figure 3

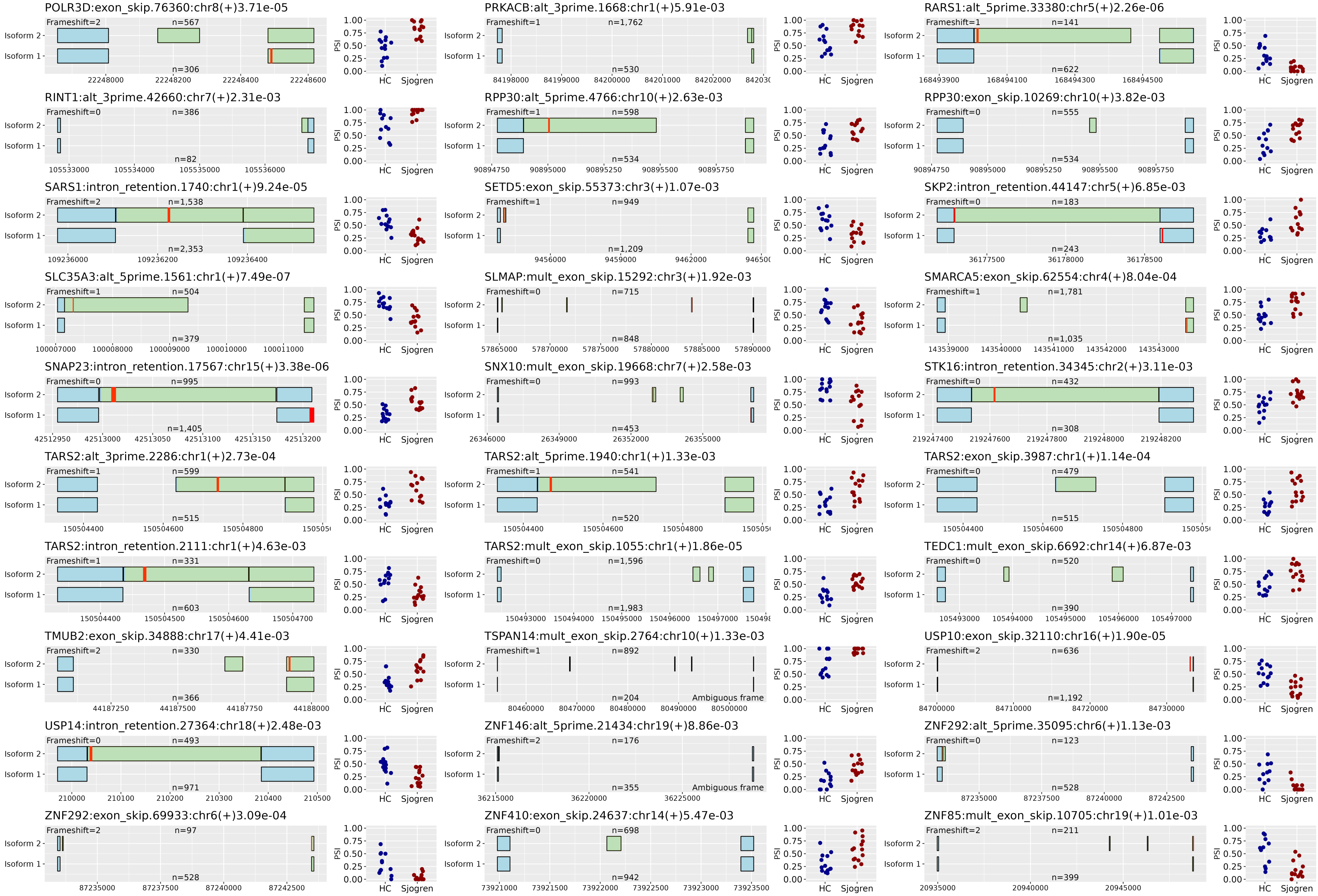

Extended Data Figure 4

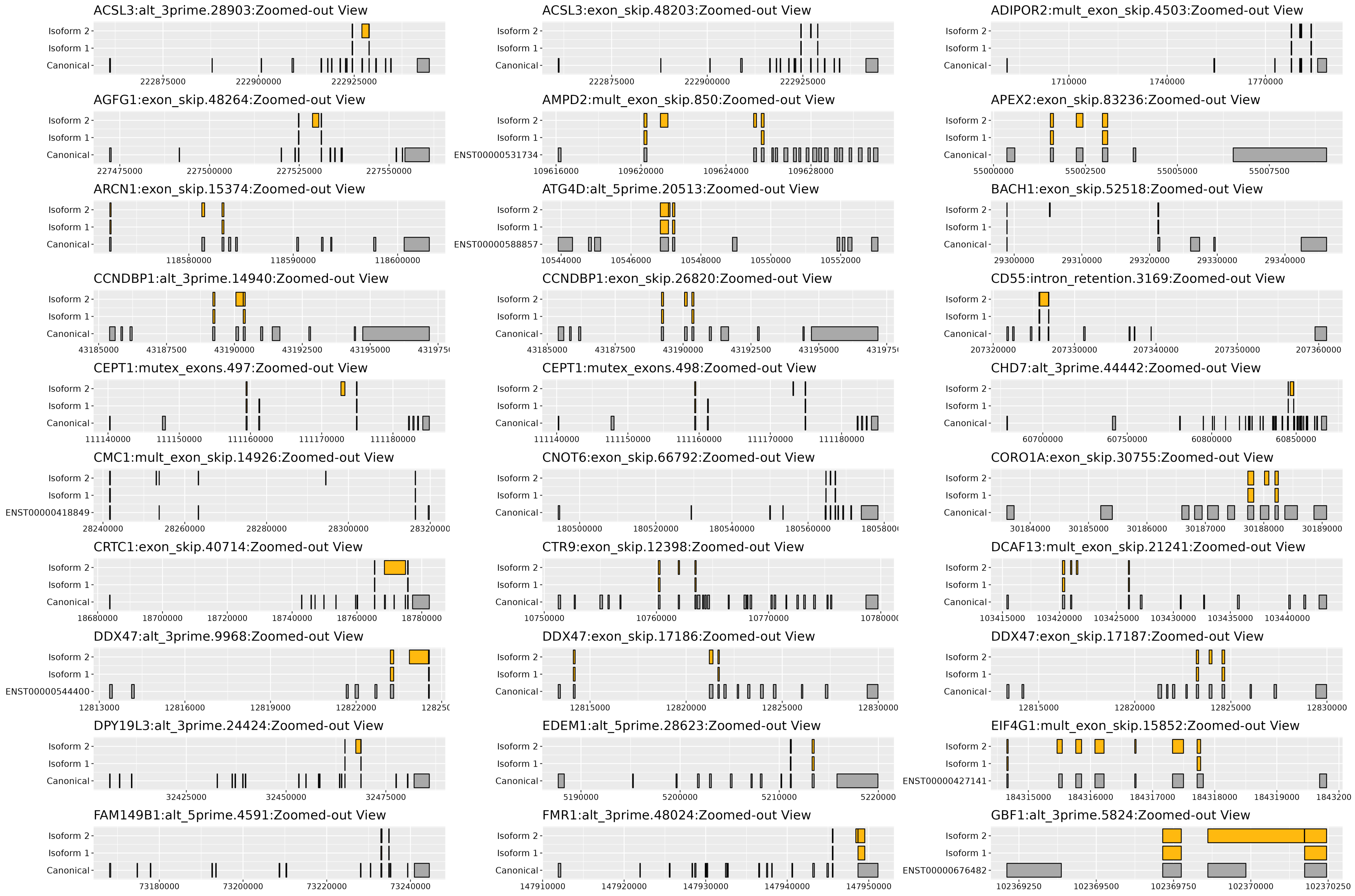

Extended Data Figure 5

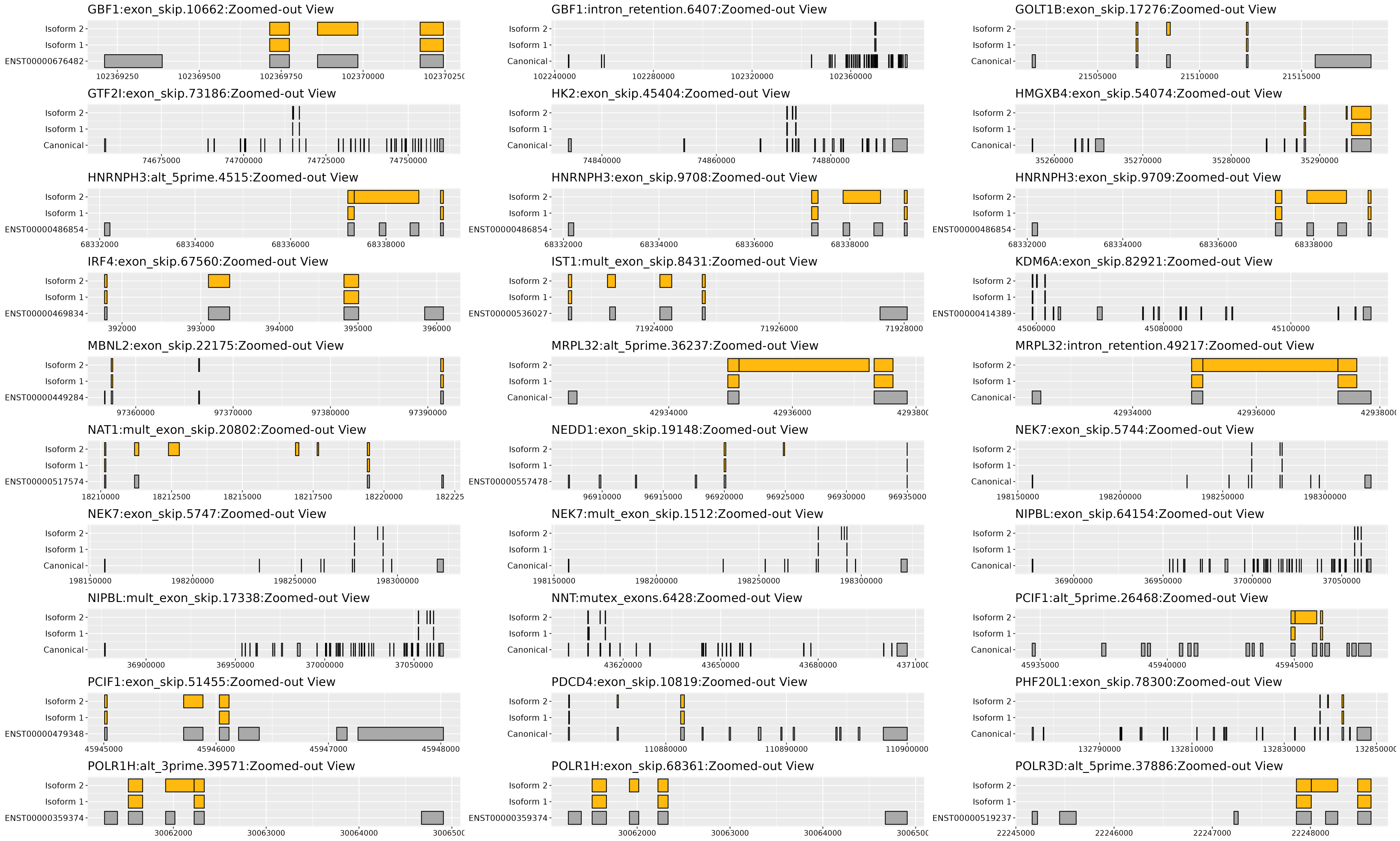

Extended Data Figure 6

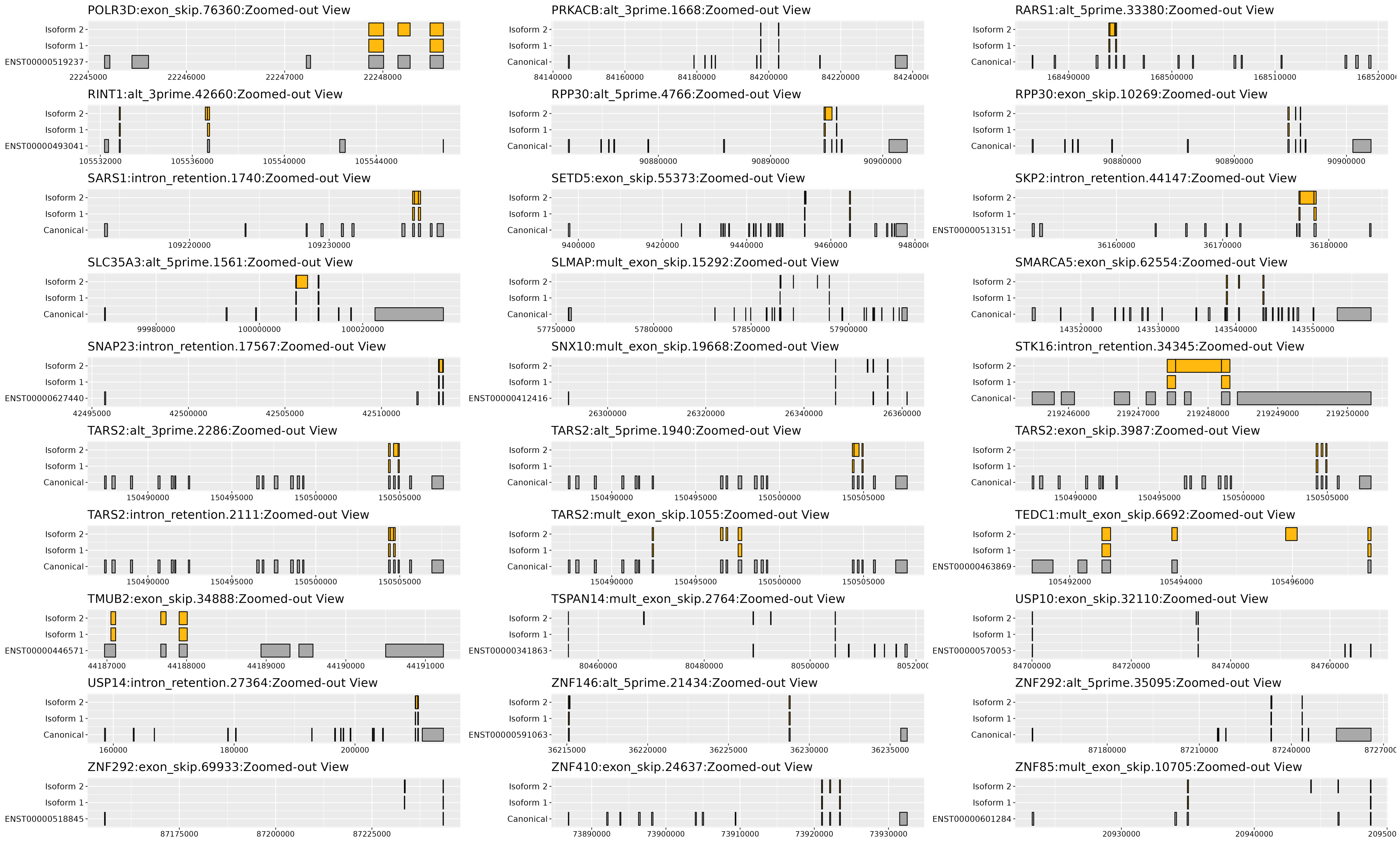

Extended Data Figure 7

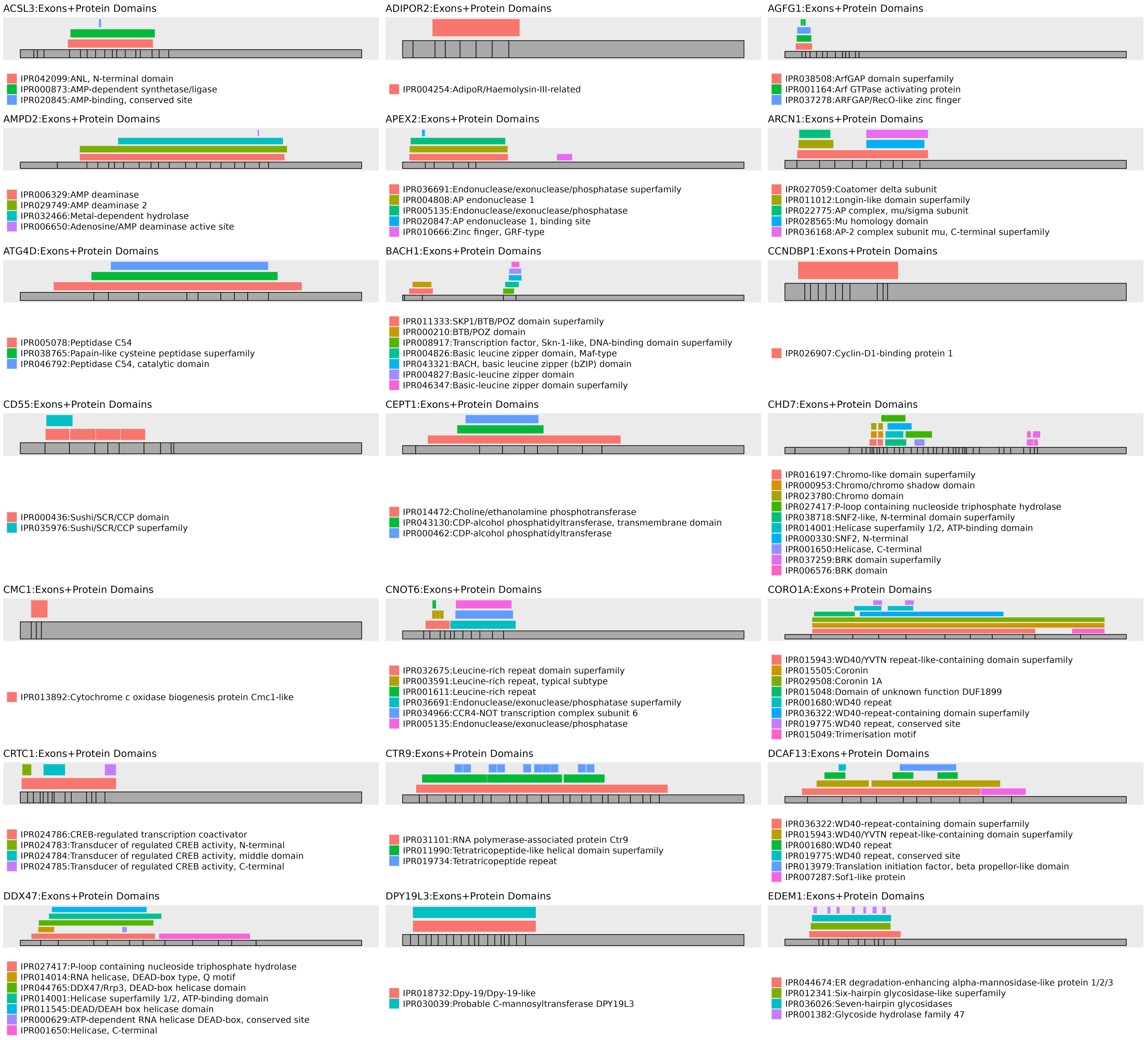

Extended Data Figure 8

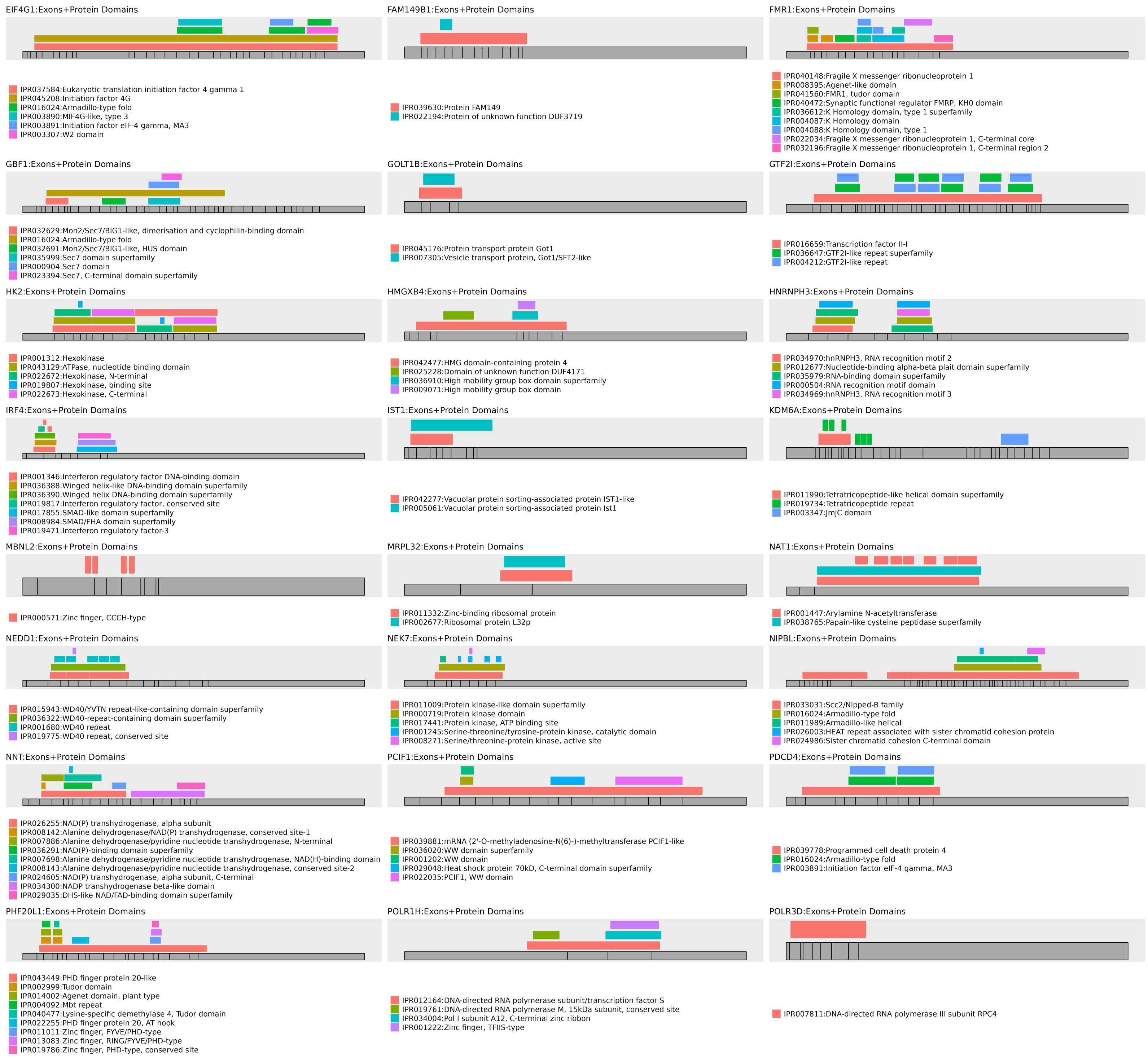

Extended Data Figure 9

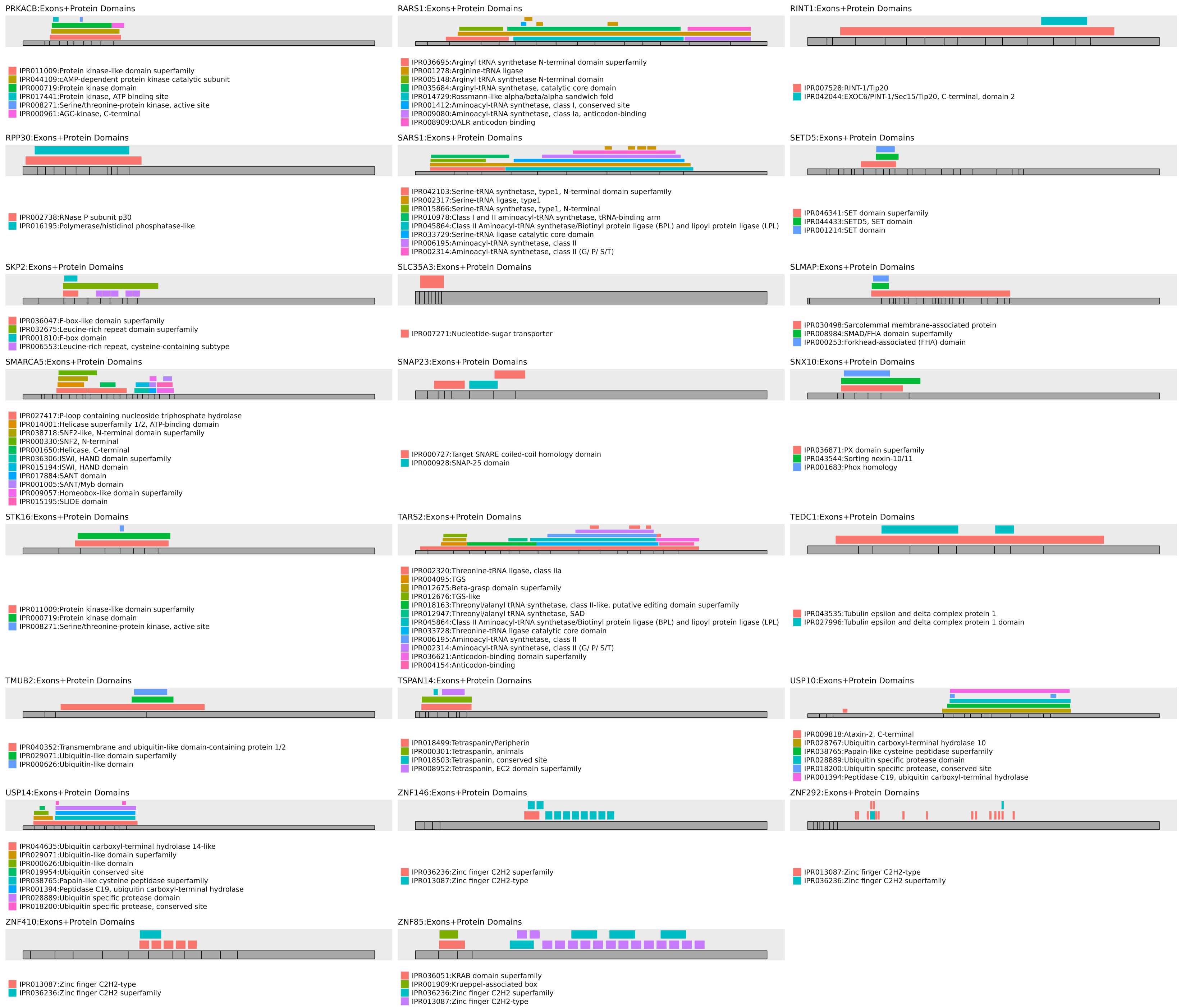

Supplement: Supplementary file 1 — Supplementary Figures. [file 41598_2023_47348_MOESM1_ESM.pdf]
